# Nutrition transition and chronic diseases in China (1990–2019): industrially processed and animal calories rather than nutrients and total calories as potential determinants of the health impact – ADDENDUM

**DOI:** 10.1017/S1368980021004146

**Published:** 2022-01

**Authors:** Anthony Fardet, Kenny Aubrun, Edmond Rock

The authors regret the omission of supplementary material in the above article. This has since been added.
